# Genome Sequence of *Marinobacter* sp. Strain MCTG268 Isolated from the Cosmopolitan Marine Diatom *Skeletonema costatum*

**DOI:** 10.1128/genomeA.00937-16

**Published:** 2016-09-08

**Authors:** Tony Gutierrez, William B. Whitman, Marcel Huntemann, Alex Copeland, Amy Chen, Nikos Kyrpides, Victor Markowitz, Manoj Pillay, Natalia Ivanova, Natalia Mikhailova, Galina Ovchinnikova, Evan Andersen, Amrita Pati, Dimitrios Stamatis, T. B. K. Reddy, Chew Yee Ngan, Mansi Chovatia, Chris Daum, Nicole Shapiro, Michael N. Cantor, Tanja Woyke

**Affiliations:** a School of Life Sciences, Heriot-Watt University, Edinburgh, United Kingdom; b Department of Microbiology, University of Georgia, Athens, Georgia, USA; c DOE Joint Genome Institute, Walnut Creek, California, USA

## Abstract

*Marinobacter* sp. strain MCTG268 was isolated from the cosmopolitan marine diatom *Skeletonema costatum* and can degrade oil hydrocarbons as sole sources of carbon and energy. Here, we present the genome sequence of this strain, which is 4,449,396 bp with 4,157 genes and an average G+C content of 57.0%.

## GENOME ANNOUNCEMENT

*Marinobacter* sp. strain MCTG268 was isolated from a laboratory culture of the marine diatom *Skeletonema costatum* (CCAP 1077/1C) by enrichment with naphthalene as the sole carbon source. Based on 16S rRNA gene sequence identity, the closest type species is *Marinobacter algicola* strain DG893, which had also been isolated from a laboratory culture of eukaryotic phytoplankton (1).

Here, we report the genome sequence of *Marinobacter* sp. strain MCTG268. Genomic DNA was sequenced through the DOE Joint Genome Institute 2014 Genomic Encyclopedia of Type Strains, Phase III study (2) using Pacific Biosciences (PacBio) technology. A Pacbio SMRTbellTM library was constructed and sequenced on the PacBio RS platform, which generated 220,290 filtered subreads totaling 696.2 Mbp. All general aspects of library construction and sequencing performed at the JGI can be found at http://www.jgi.doe.gov. The raw reads were assembled using HGAP (version: 2.1.1) (3). The final draft assembly produced 14 scaffolds containing 14 contigs totaling 4.4 Mbp in size and input read coverage of 222.5×.

Project information is available in the Genomes Online Database (4). Genes were identified using Prodigal (5), followed by a round of manual curation using GenePRIMP (6) as part of JGI’s microbial annotation pipeline (7). The predicted coding sequences (CDSs) were translated and used to search the National Center for Biotechnology Information (NCBI) nonredundant, UniProt, TIGRFam, Pfam, KEGG, COG, and InterPro databases. The tRNAScanSE tool (8) was used to find tRNA genes, whereas rRNA genes were found by searches against models of the rRNA genes built from SILVA (9). Other noncoding RNAs, such as the RNA components of the protein secretion complex and the RNase P, were identified by searching the genome for the corresponding Rfam profiles using INFERNAL (http://infernal.janelia.org). Additional gene prediction analysis and manual functional annotation was performed within the Integrated Microbial Genomes–Expert Review (IMG ER) platform (http://img.jgi.doe.gov) developed by the Joint Genome Institute, Walnut Creek, CA, USA (10).

The complete genome sequence length was 4,449,396 bp with a G+C content of 57.0%. The genome contains 4,157 genes (4,083 protein-coding genes) with functional predictions for 3,388 of them. A total of 74 RNA genes were detected. Other genes, characteristic for the genus, are given in the IMG database (10).

### Accession number(s).

The draft genome sequence of *Marinobacter* sp. strain MCTG268 obtained in this study was deposited in GenBank as part of BioProject no. PRJNA224116, with individual genome sequences submitted as whole-genome shotgun projects under the accession no. JQMK00000000.
